# Development and Characterization of Polymeric Microspheres for Controlled Release Protein Loaded Drug Delivery System

**DOI:** 10.4103/0250-474X.42978

**Published:** 2008

**Authors:** S. Ravi, K. K. Peh, Yusrida Darwis, B. Krishna Murthy, T. Raghu Raj Singh, C. Mallikarjun

**Affiliations:** School of Pharmaceutical Sciences, Universiti Sains Malaysia, Gelugor, Pulau Penang, 11800, Malaysia

**Keywords:** Bovine serum albumin, poly(D,L-lactide-co-glycolide), w/o/w double emulsion, microspheres, *in vitro* release

## Abstract

The aim of the present work was to investigate the preparation of microspheres as potential drug carriers for proteins, intended for controlled release formulation. The hydrophilic bovine serum albumin was chosen as a model protein to be encapsulated within poly(D,L-lactide-co-glycolide) (50:50) microspheres using a w/o/w double emulsion solvent evaporation method. Different parameters influencing the particle size, entrapment efficiency and *in vitro* release profiles were investigated. The microspheres prepared with different molecular weight and hydrophilicity of poly(D,L-lactide-co-glycolide) polymers were non porous, smooth surfaced and spherical in structure under scanning electron microscope with a mean particle size ranging from 3.98 to 8.74 μm. The protein loading efficiency varied from 40 to 71% of the theoretical amount incorporated. The *in vitro* release profile of bovine serum albumin from microspheres presented two phases, initial burst release phase due to the protein adsorbed on the microsphere surface, followed by slower and continuous release phase corresponding to the protein entrapped in polymer matrix. The release rate was fairly constant after an initial burst release. Consequently, these microspheres can be proposed as new controlled release protein delivery system.

In the last twenty years or so, controlled release technology has emerged as an important new field in the development of pharmaceutical dosage forms^1^. At present, protein delivery is a very promising area of research, due to the recognized necessity of improving the *in vivo* efficacy of the currently being developed therapeutic as well as antigenic proteins^2^. Generally, they have short plasma half-lives, are incapable of diffusing through biological membranes, are unstable in the gastrointestinal (GI) tract, and also have low bioavailabilities due to their large molecular weight and high aqueous solubility^3^,^4^. Thus, development of sustained release parenteral dosage forms for protein delivery becomes necessary to overcome the problems of patient incompliance and inconvenience where the therapy is required over months and years in chronic diseases^5^.

Biodegradable polymeric drug delivery systems (DDS) such as those based on aliphatic polyesters, polylactic acid (PLA), polyglycolic acid (PGA) and poly(D,L-lactide-co-glycolide) (PLGA) microspheres have been studied extensively during the past three decades as a formulation approach to protect encapsulated drugs from degradation, enhance bioavailability and sustain drug release^6^,^7^. Biodegradable DDS have been used to deliver a variety of therapeutic substances such as proteins, peptides, NSAIDs, antibiotics and anticancer drugs in recent years because of their biocompatibility and degradation *in vivo*, to toxicologically acceptable lactic and glycolic acids which are further eliminated by the normal metabolic pathways and approved by US FDA.

Although a wide range of microencapsulation techniques have been developed for the preparation of sustained release delivery, the method of preparation has much influence on the properties of microspheres and therefore the desired properties should be kept in mind during the selection of a particular method of preparation.

A water-in-oil-in-water (w/o/w) double emulsion technique has been developed for the encapsulation of hydrophilic drugs, in order to improve the loading efficiency of microspheres and also to minimize their exposure to organic solvents during the manufacturing process and thus, minimize any loss of bioactivity^8^.

The objective of the present study was to prepare and characterize protein loaded biodegradable polymeric microspheres. Bovine serum albumin has been selected as the model protein since it is one of the widely studied proteins with a relatively high molecular weight, most abundantly available and of low cost. This protein was encapsulated into different molecular weight and hydrophilicity of PLGA polymers, by double emulsion solvent evaporation technique, for the delivery of proteins over an extended period of time.

## MATERIALS AND METHODS

Bovine serum albumin (BSA, MW 66 430 Da) and sodium azide were purchased from Sigma (St. Louis, USA). PLGA (50:50) polymers, Resomer^®^ RG 502 H (MW 13 600), RG 503 H (MW 35 700), RG 502 (MW 17 200) and RG 504 (MW 48 300) were purchased from Boehringer Ingelheim (Ingelheim, Germany). Polyvinyl alcohol (PVA) (MW 1 40 000 Da) and mannitol were obtained from BDH Laboratory Supplies (Poole, England). Potassium dihydrogen phosphate, disodium hydrogen phosphate, sodium dodecyl sulphate (SDS), sodium hydroxide, Tween 80 and dichloromethane (DCM) were purchased from R & M Chemicals (Essex, U.K.). Sodium chloride was purchased from Hmbg Chemicals (Hamburg, Germany). Hydrochloric acid was purchased from Lab Scan (Bangkok, Thailand). All the solvents used were of analytical grade and the materials were used as received.

### Preparation of BSA loaded PLGA microspheres:

BSA loaded PLGA microspheres were prepared by a double emulsion solvent evaporation technique as previously described by Rafati *et al*^9^ and conveniently modified. Briefly, PLGA was dissolved in 10 ml dichloromethane and emulsified with BSA aqueous solution using a homogenizer (Ultraturrax T-18, IKA Works Inc, Wilmington, USA) at a speed of 14 000 rpm for 5 min to form a primary emulsion (w/o). The polymer concentration in organic phase was 60 mg/ml and the BSA to polymer weight ratio 1:15. This primary emulsion was rapidly transferred into 25 ml of aqueous solution containing 3% w/v of PVA as an emulsifier and homogenized for 5 min at the same speed to produce a double (w/o/w) emulsion. The resultant double emulsion was magnetically stirred for 6 h at ambient room temperature at 250 rpm to evaporate dichloromethane. The hardened microspheres were isolated by centrifugation at 2185xg for 15 min (Sigma 2-15, USA) and washed thrice using distilled water. Mannitol (1% w/v) was added before lyophilization (Labconco, USA) to prevent aggregation of microspheres. The microspheres were stored at 4° until the time of evaluation.

### Effect of formulation and processing variables:

The effect of the following formulation and process variables on the characterization of microspheres was investigated. The effect of different speeds of magnetic stirrer with the length of 4.0×0.75 cm (250, 750, 1250 rpm) and Ultraturrax homogenizer (6000, 10 000, 14 000 rpm) were studied to achieve desired particle size in the range of 1-5 μm. The PLGA polymer, RG 502 was used for the preparation of microspheres. The effect of organic solvents with different water solubility such as acetone, ethyl acetate and dichloromethane on the particle size and entrapment efficiency of microspheres was studied. The PLGA polymer, RG 502 microspheres were prepared with the speed of 14 000 rpm using Ultraturrax homogenizer. The effect of molecular weight and hydrophilicity of PLGA (50:50) biodegradable polymers on the particle size, morphology, entrapment efficiency and *in vitro* release profiles of microspheres was investigated. PLGA (50:50) polymers, Resomer^®^ RG 502, RG 502 H, RG 503 H and RG 504 were used for this study.

### Characterization of microspheres:

The particle size of microspheres was estimated using Malvern Mastersizer (Mastersizer S, Malvern Instruments Limited, U.K.). The freeze dried microspheres were dispersed by bath sonication in saline medium (0.9% NaCl) containing a surfactant (0.1% Tween 80) to prevent aggregation before samples examination. Samples were analyzed in triplicate. The particle size was expressed as the mean volume diameter in μm.

The morphological examination of microspheres containing BSA was performed using SEM (SEM, Leica Cambridge S360, UK). The formulations which were used for the study of effect of different molecular weight and hydrophilicity of PLGA polymers were subjected to SEM analysis. For the shape and surface analysis, the freeze dried microspheres were mounted onto aluminum stub using double-sided adhesive tape and then sputter coated with a thin layer of gold under argon atmosphere (Emitech K750, Kent, UK) before examination. The coated specimen was then examined under the microscope at an acceleration voltage of 2 kV and photographed.

The BSA content of microspheres was analyzed using hydrolysis technique as previously described by Igartua *et al*^10^. Briefly, 15 mg of lyophilized microspheres were digested with 5 ml of 0.1 M NaOH containing 5% w/v SDS and stirred for 15 h at ambient temperature until a clear solution was obtained. Sodium hydroxide catalyzes the hydrolysis of the polymer and SDS ensures the complete solubilization of the protein during the polymer hydrolysis. The resulting clear solution was then neutralized to pH 7 by addition of 1 M HCl and centrifuged at 1726×g for 15 min. The samples were analyzed in triplicate for each batch of microspheres using UV-Visible Spectrophotometer (Hitachi 2000, Japan) at 215 nm. The encapsulation efficiency was expressed as the ratio of actual to theoretical BSA content. The entrapment efficiency was calculated using following equation, Entrapment efficiency (%) = (Weight of BSA in microspheres/Weight of BSA fed initially)×100.

*In vitro* release studies were carried out by suspending 100 mg of microspheres in 60 ml of phosphate buffered saline (PBS, pH 7.4) containing 0.02% sodium azide as bacteriostatic agent and 0.01% Tween 80 to prevent the microspheres from aggregation in the dissolution medium in stoppered flasks. The flasks were placed in a reciprocal shaking water bath maintained at 37±0.5° at a speed of 60 cycles/min. At predetermined time intervals of 2, 12, 24, 72, 120 and 168 h, samples were collected and centrifuged at 1726×g for 15 min. The supernatant was assayed for the protein release using UV-Vis spectrophotometer at a detection wavelength of 215 nm. The collected amount of supernatant was replaced with fresh PBS to maintain sink conditions. The percentage of protein release at different intervals was calculated by using a freshly prepared calibration curve using the standard samples which were run along with test samples. Release experiments were done independently in triplicate for each batch of microspheres.

### Statistical analysis:

The results are presented as mean±standard deviation. The particle size and entrapment efficiency of BSA loaded microspheres were treated statistically using one-way analysis of variance (ANOVA). When there was a statistically significant difference, a post-hoc Tukey-HSD (Honestly Significant Difference) test was performed. A statistically significant difference was considered at p<0.05.

## RESULTS AND DISCUSSION

For the formulation of microspheres from the biodegradable polymer matrix it is essential to select an encapsulation process which fulfils the requirements of an ideal controlled release system such as free flowing microspheres with desired size, high yield and entrapment efficiency, low burst release with preferred release profiles, stability of the encapsulated protein and a major requirement is syringeability of microspheres through hypodermic needles. The biological activity of the encapsulated protein also should be maintained during the process of microsphere formulation^11^.

The selection of a particular encapsulation method is usually determined by solubility of the protein and the coating polymer; it also has utmost importance for the efficient entrapment of active substance. In this study, double emulsion solvent evaporation technique was adopted for the efficient incorporation of BSA in the biodegradable polymeric microspheres due to the solubility of protein in aqueous phase and the organic phase or oil phase acts as a barrier between the two aqueous compartments, preventing the diffusion of the active material toward the external aqueous phase^12^. It is also known to be superior to other encapsulation methods in terms of stability of proteins due to minimize exposure to organic solvent during preparation of microspheres. Encapsulation by the solvent evaporation technique involves two major steps, emulsification of an organic solvent containing dissolved polymer and dissolved/dispersed protein in an excess amount of aqueous continuous phase to form a stable droplets and the subsequent removal of organic solvent from the droplets of the second emulsion.

Particle size is one of the most important characteristics of the microspheres. In the preliminary study of this work, effect of different speeds of magnetic stirrer and homogenizer were studied to achieve smaller size of microspheres. The particle size of microspheres decreased from 80.85 to 62.04 μm and 15.47 to 4.56 μm with the increase of stirring speed of magnetic stirrer from 250 to 1250 rpm and homogenizer speed from 6000 to 14 000 rpm, respectively. The results are shown in Table 1.

**TABLE 1:** EFFECT OF STIRRING SPEED ON PARTICLE SIZE OF BSA LOADED PLGA MICROSPHERES

Formulation	BSA (mg)	PLGA (RG 502) (mg)	Stirring speed (rpm)	Particle size (μm) (Mean±SD, n=3)
F1	40	600	250*	80.85±0.34
F2	40	600	750*	74.23±0.97
F3	40	600	1250*	62.04±0.49
F4	40	600	6000**	15.47±0.29
F5	40	600	10 000**	11.53±0.31
F6	40	600	14 000**	4.56±0.09

The particle size of BSA loaded PLGA microspheres prepared with different stirring speeds of magnetic stirrer (*) and Ultraturrax homogenizer speed. (**)

Size of microspheres was determined by the stirring speed. Stirring speed was parameter of primary importance in the emulsification step because it provides the energy to disperse the oil phase in aqueous phase. Our experimental results demonstrated that mean particle size of microspheres was inversely proportional to stirring speed; consequently increase in stirring speed decreased the size of microspheres because the second emulsion was broken up into smaller droplets at a higher input power in accordance with the study of Yang *et al*^13^. Thus, the stirring speed was optimized in order to obtain a desired size of microspheres in the range of 1-5 μm because the size of microspheres plays a crucial role in determination of the uptake of the encapsulated protein by immune system, *in vivo* distribution of the particles after administration and syringeability through hypodermic needles^2^. There was a statistically significant difference (p<0.05) in the size of microspheres prepared with different speeds of magnetic stirrer and homogenizer. A homogenizer stirring speed at 14 000 rpm was chosen for further study.

The selection of suitable organic solvent is critical in developing a successful formulation of biodegradable microspheres containing protein. The parameters, solvent's miscibility/solubility in water and its ability to dissolve the polymer should be considered while choosing organic solvent. In this process, acetone, ethyl acetate and dichloromethane organic solvents with percentage of solubility of 100, 8.7 and 1.6% w/w in water respectively were used.

The results of particle size and entrapment efficiency of microspheres prepared with different organic solvents are shown in Table 2. The size of microspheres was highly influenced by the water solubility of organic solvent. The mean particle size of microspheres with acetone, ethyl acetate and dichloromethane were 11.43, 9.16 and 4.56 μm, respectively. The larger size of microspheres were formed with the higher water solubility of organic solvent because of the irregular polymer agglomeration upon emulsification due to rapid solvent exchange into aqueous phase^1^. The use of dichloromethane instead of acetone and ethyl acetate resulted in particles which were smaller in size. This was probably because dichloromethane is a better solvent for PLGA than acetone and ethyl acetate, leading to the formation of denser microspheres. There was a statistically significant difference (p<0.05) in the size of microspheres prepared with different organic solvents.

**TABLE 2:** EFFECT OF ORGANIC SOLVENT ON PARTICLE SIZE AND ENCAPSULATION EFFICIENCY OF BSA LOADED PLGA MICROSPHERES

Formulation	Organic solvent	Particle size (μm) (Mean±SD, n=3)	Encapsulation efficiency (%) (Mean±SD, n=3)
F6	Dichloromethane	4.56±0.09	40.14±1.27
F7	Ethyl acetate	9.16±0.06	29.93±4.17
F8	Acetone	11.43±0.38	29.57±4.87

The particle size and encapsulation efficiency of BSA loaded PLGA microspheres prepared with different water solubility organic solvents (Dichloromethane, Ethyl acetate and Acetone).

The successful entrapment of protein was dependent to a large extent on the type of organic solvent used. The entrapment efficiency of microspheres prepared with acetone, ethyl acetate and dichloromethane were 29.57, 29.93 and 40.14%, respectively. Bodmeier and McGinity found from the experiments that the rate of polymer precipitation from the organic solvent phase was strongly affected by the rate of diffusion of the organic solvent into the aqueous phase^1^. Organic solvents of low water solubility resulted in slow polymer precipitation which facilitated complete partitioning of the drug into the aqueous phase, resulting in empty microspheres^14^. On the other hand, highly water-miscible solvents did not form droplets but large irregular polymer agglomerates upon emulsification due to rapid solvent exchange it leads to non uniform and poor encapsulation efficiency. The relatively high water miscible organic solvents acetone and ethyl acetate did not encapsulate the protein efficiently and produced larger particle size of microspheres compared to dichloromethane which is relatively a low water miscible solvent. The only organic solvent which could successfully encapsulate higher amount of protein (40.14%) with smaller size of microspheres under the selected experimental conditions was dichloromethane. This might be due to the optimum solubility of dichloromethane in water. There was statistically significant difference (p <0.05) among the formulations prepared with acetone and dichloromethane as well as ethyl acetate and dichloromethane. These results indicated that dichloromethane is a good solvent for the formation of microspheres with higher entrapment efficiency due to its desirable physical properties such as extremely low solubility in water, ability to dissolve large amounts of polymer and required the lowest heat of evaporation. The organic solvent, dichloromethane was selected for further study.

The influence of molecular weight and hydrophilicity of PLGA polymers on particle size, morphology, entrapment efficiency and *in vitro* release profiles of microspheres were studied. PLGA (50:50) polymers, Resomer^®^ RG 502 H, RG 502, RG 503 H and RG 504 were used for this study. The results of particle size and entrapment efficiency of the protein loaded microspheres are shown in Table 3.

**TABLE 3:** EFFECT OF MOLECULAR WEIGHT AND HYDROPHILICITY OF PLGA POLYMERS ON PARTICLE SIZE AND ENCAPSULATION EFFICIENCY OF MICROSPHERES

Formulation	PLGA (50:50) polymer	Particle size (μm) (Mean±SD, n=3)	Encapsulation efficiency (%) Mean±SD, n=3)
F6	RG 502	4.56±0.09	40.14±1.27
F9	RG 502 H	3.98±0.17	48.33±2.43
F10	RG 503 H	6.53±0.07	57.72±2.51
F11	RG 504	8.74±0.69	71.05±4.77

The particle size and encapsulation efficiency of BSA loaded PLGA microspheres prepared with RG 502, RG 502 H, RG 503 H and RG 504.

The mean particle size of PLGA microspheres, RG 502 H, RG 502, RG 503 H and RG 504 were 3.98, 4.56, 6.53 and 8.74 μm, respectively. The particle size of the microspheres was mainly affected by the molecular weight of the polymers. At a constant solvent volume, the viscosity of the polymeric solution was proportional to the molecular weight of the polymer. Low viscosity polymer solution could be dispersed in the external aqueous phase to a greater extent than high viscosity polymer solution and hence reduced the microspheres to smaller sizes. There was no statistically significant difference (p>0.05) in the particle size of microspheres between RG 502 and RG 502 H. This might be due to the small difference in their molecular weight of polymers, which could influence the size of microspheres. There was a statistically significant difference (p<0.05) in the size of microspheres among the polymers, RG 502 H and RG 503 H; RG 502 H and RG 504; RG 502 and RG 503 H; RG 502 and RG 504 as well as RG 503 H and RG 504.

**Fig. 1:**
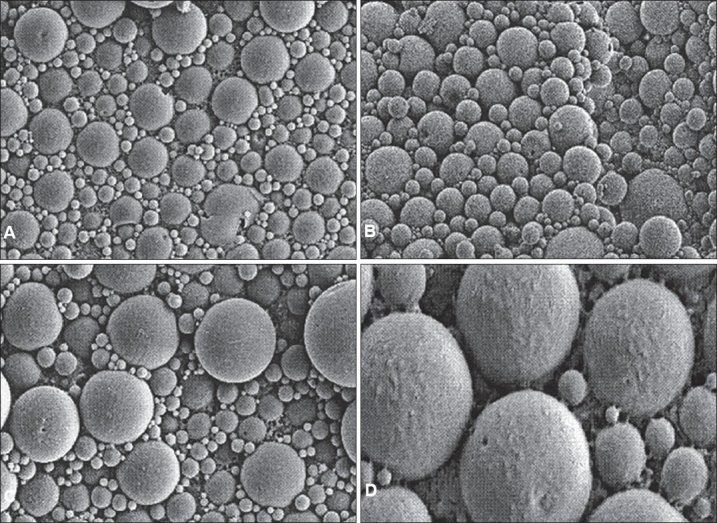
SEM of BSA loaded microspheres prepared with different molecular weight and hydrophilicity of PLGA (50:50). Scanning electron micrographs of BSA containing PLGA microspheres formulated by double emulsion technique: (A) RG 502; (B) RG 502 H; (C) RG 503 H and (D) RG 504 at a magnification of 5.01 K X.

**Fig. 2:**
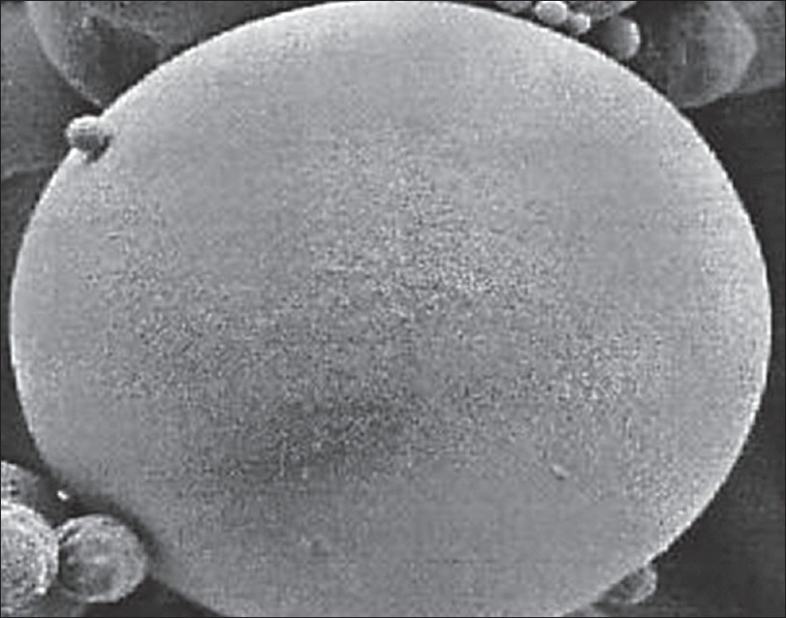
SEM of surface characteristics of BSA loaded PLGA microspheres. Scanning electron micrograph of the surface characteristics of BSA containing PLGA microspheres at a magnification of 4.02 K X.

The morphological examination of the BSA loaded microspheres was performed by the SEM. The BSA loaded PLGA microspheres were spherical in shape and had a smooth surface without pores or cavities which could affect the release of encapsulated protein. The same appearance was observed for all formulations, independent of the type of polymer. The SEM photographs are shown in fig. 1 and 2.

**Fig. 3:**
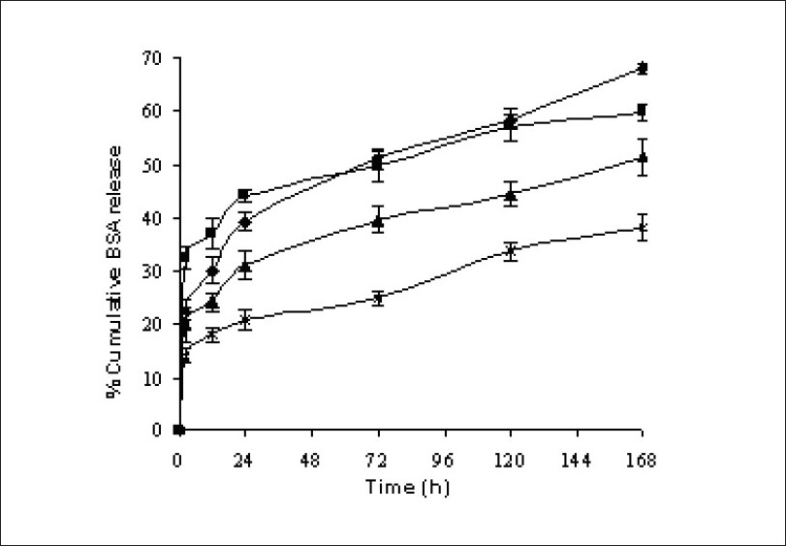
*In vitro* release of BSA loaded PLGA microspheres from the formulations F6, F9, F10 and F11. *In vitro* release profiles of BSA loaded microspheres formulated with different molecular weights of PLGA 50:50 polymers, RG 502 H (–♦–), RG 502 (–■–), RG 503 H (–▲–) and RG 504 (–×–). Mean ± SD, n=3.

The encapsulation efficiency of protein loaded PLGA polymers, RG 502 H, RG 502, RG 503 H and RG 504 were 48.33, 40.14, 57.72 and 71.05%, respectively. The encapsulation efficiency of protein was highly influenced by the molecular weight and hydrophilicity of PLGA polymers. BSA encapsulation efficiency was higher for RG 504 and RG 503 H compared to RG 502 and RG 502 H. This might be attributed to the higher viscosity of polymeric solution as a result of high molecular weight of the polymer which could prevent the transfer of protein from internal aqueous phase to external aqueous phase and/or pronounced molecular weight dependent attraction forces between protein and polymer also caused the entrapment of more protein. The higher encapsulation efficiency was observed for the hydrophilic polymer RG 502 H compared to RG 502. This might be due to the strong affinity between positively charged amino acid groups in protein and negatively charged free carboxyl end groups in the polymer chain. There was a statistically significant difference (p<0.05) in the entrapment efficiency of protein among the PLGA polymers. The *in vitro* release profiles of microspheres are intended to assist in predicting the ultimate behaviour of a given microsphere formulation. The release of BSA from the microspheres showed a biphasic profile as shown in fig. 3. The microspheres showed an initial rapid release of a certain amount of protein which was deposited on the surface of the microspheres, followed by a slow and continuous release which corresponds to release of protein entrapped in the microspheres.

The polymer with terminal carboxylic groups, RG 502 H showed low initial burst release compared to RG 502. This could be due to the hydrophilicity of polymer which causes favourable interaction between free carboxylic end groups in the polymer chains and protein. The percentage release of protein from microspheres in one week was 68.13, 59.87, 51.66 and 38.31% for RG 502 H, RG 502, RG 503 H and RG 504, respectively. This might be attributed to the molecular weight of the polymer. The low molecular weight of PLGA polymer degraded into small fragments which are soluble in aqueous release medium, thus leading to faster erosion of the microspheres. During erosion, the polymer matrix becomes more and more hydrophilic, allowing more water to penetrate, thereby enhancing polymer degradation and thus, protein release^15^.

In conclusion, protein loaded PLGA microspheres were prepared successfully using the double emulsion solvent evaporation technique. The size of the microspheres was influenced by the stirring speed, organic solvent and molecular weight of polymers. The microspheres were spherical in shape with a smooth and non porous surface. The water miscibility of organic solvents plays a role in successful entrapment of proteins. The *in vitro* protein release study from PLGA microspheres proved that the present microspheres had the properties of an ideal sustained release formulation. Furthermore, the present microspheres are attractive for parenteral application because of their smaller size and biodegradability.

## References

[1] Bodomeier R, McGinity JW (1988). Solvent selection in the preparation of poly(D,L-lactide) microspheres prepared by the solvent evaporation method. Int J Pharm.

[2] Blanco MD, Alonso MJ (1997). Development and characterization of protein – loaded poly (lactide-co-glycolide) nanospheres. Eur J Pharm Biopharm.

[3] Ravivarapu HB, Burton K, Deluca PP (2000). Polymer and microsphere blending to alter the release of a peptide from PLGA microspheres. Eur J Pharm Biopharm.

[4] Sanders LM (1990). Drug delivery systems and routes of administration of peptide and protein drugs. Eur J Drug Metab Pharmacokinet.

[5] Feirong K, Jagdish S (2001). Effect of additives on the release of a model protein from PLGA microspheres. AAPS PharmsciTech.

[6] Okada H, Toguchi H (1995). Biodegradable microspheres in drug delivery. Crit Rev Ther Drug Carrier Syst.

[7] Nitsch MJ, Banakar UV (1994). Implantable drug delivery. J Biomater Appl.

[8] Yan C, Resau JH, Hewetson J, West M, Rill WL, Kende M (1994). Characterization and morphological analysis of protein-loaded poly(lactide-co-glycolide) microparticles prepared by water-in-oil-in-water emulsion technique. J Control Release.

[9] Rafati H, Coombes AG, Adler J, Holland J, Davis SS (1997). Protein loaded poly (DL-lactide-co-glycolide) microparticles for oral administration: Formulation, structural and release characteristics. J Control Release.

[10] Igartua M, Hernandez A, Esquisabel A, Gascon AR, Calvo MB, Pedraz JL (1998). Stability of BSA encapsulated into PLGA microspheres using PAGE and capillary electrophoresis. Int J Pharm.

[11] Sinha VR, Trehan A (2003). Biodegradable microspheres for protein delivery. J Control Release.

[12] Hincal AA, Calis S, Wise DL (2000). Microsphere preparation by solvent evaporation method. Handbook of pharmaceutical controlled release technology.

[13] Yang YY, Chung TS, Ng NP (2001). Morphology, drug distribution, and *in vitro* release profiles of biodegradable polymeric microspheres containing protein fabricated by double-emulsion solvent extraction/evaporation method. Biomaterials.

[14] O'Donell PB, McGinity JW (1997). Preparation of microspheres by the solvent evaporation technique. Adv Drug Del Rev.

[15] Blanco D, Alonso MJ (1998). Protein encapsulation and release from poly(lactide-co-glycolide) microspheres: Effect of the protein and polymer properties and of the co-encapsulation of surfactants. Eur J Pharm Biopharm.

